# Ensemble Learning Based on Hybrid Deep Learning Model for Heart Disease Early Prediction

**DOI:** 10.3390/diagnostics12123215

**Published:** 2022-12-18

**Authors:** Ahmed Almulihi, Hager Saleh, Ali Mohamed Hussien, Sherif Mostafa, Shaker El-Sappagh, Khaled Alnowaiser, Abdelmgeid A. Ali, Moatamad Refaat Hassan

**Affiliations:** 1 Department of Computer Science, College of Computers and Information Technology, Taif University, P.O. Box 11099, Taif 21944, Saudi Arabia; 2 Faculty of Computers and Artificial Intelligence, South Valley University, Hurghada 84511, Egypt; 3 Department of Computer Science, Faculty of Science, Aswan University, Aswan 81528, Egypt; 4 Faculty of Computer Science and Engineering, Galala University, Suez 34511, Egypt; 5 Information Systems Department, Faculty of Computers and Artificial Intelligence, Benha University, Banha 13518, Egypt; 6 College of Computer Engineering and Sciences, Prince Sattam Bin Abdulaziz University, Al Kharj 11942, Saudi Arabia; 7 Faculty of Computers and Information, Minia University, Minia 61519, Egypt

**Keywords:** machine learning, deep learning, ensemble learning, heart disease

## Abstract

Many epidemics have afflicted humanity throughout history, claiming many lives. It has been noted in our time that heart disease is one of the deadliest diseases that humanity has confronted in the contemporary period. The proliferation of poor habits such as smoking, overeating, and lack of physical activity has contributed to the rise in heart disease. The killing feature of heart disease, which has earned it the moniker the “silent killer,” is that it frequently has no apparent signs in advance. As a result, research is required to develop a promising model for the early identification of heart disease using simple data and symptoms. The paper’s aim is to propose a deep stacking ensemble model to enhance the performance of the prediction of heart disease. The proposed ensemble model integrates two optimized and pre-trained hybrid deep learning models with the Support Vector Machine (SVM) as the meta-learner model. The first hybrid model is Convolutional Neural Network (CNN)-Long Short-Term Memory (LSTM) (CNN-LSTM), which integrates CNN and LSTM. The second hybrid model is CNN-GRU, which integrates CNN with a Gated Recurrent Unit (GRU). Recursive Feature Elimination (RFE) is also used for the feature selection optimization process. The proposed model has been optimized and tested using two different heart disease datasets. The proposed ensemble is compared with five machine learning models including Logistic Regression (LR), Random Forest (RF), K-Nearest Neighbors (K-NN), Decision Tree (DT), Naïve Bayes (NB), and hybrid models. In addition, optimization techniques are used to optimize ML, DL, and the proposed models. The results obtained by the proposed model achieved the highest performance using the full feature set.

## 1. Introduction

Heart disease is among the most common illnesses that persisted in the past and have increased and spread in our present. The reasons for the increase in its rates are varied, especially in our modern age. Diabetes, hypertension, cholesterol, erratic heartbeat, and many more clinical signs are some biological markers and risk factors that are needed to diagnose heart disease. World Health Organization (WHO) claims that one of the main and highly-ranked causes of death worldwide is heart disease, which can have several forms such as ischemic, hypertensive, and vascular heart disease [1], and it has been shown that cardiovascular illnesses kill 17.9 million patients each year. In addition, unhealthy behavior that results in being overweight, obesity, and hypertension raises the risk of heart disease [1]. In addition, the heart is one of the essential organs of the human body. It is primarily responsible for the continuity of pumping the blood needed for the work of the rest of the human body. However, it is difficult for the heart to maintain the same efficiency throughout a person’s life. The heart is exposed to many problems that can occur because of several different reasons, such as bad health and nutritional habits or aging [2]. Therefore, finding methods and techniques that allow for the early detection or even prediction of potential heart problems has become inevitable. This can help doctors and healthcare organizations to reduce the problems and complications of the disease.

Artificial intelligence (AI) based on machine learning (ML) and deep learning (DL) has conducted key roles in evaluating medical data to assist in illness diagnosis to determine the appropriate treatment. It is used to find patterns automatically from the clinical data and then reason about clinical data to predict the early risk for patients such as heart disease [3], cancer disease [4,5], and COVID-19 [6,7]. Recently, deep learning algorithms such LSTM, GRU, CNN, and hybrid models of these algorithms have played an important role in strengthening and enhancing the level of heart disease prediction using various layers that could collect deeper features [8,9,10,11]. Recently, authors have used ensemble learning to enhance the performance of these models in the healthcare domain [12]. Ensemble learning combines the decisions of various base classifiers using many techniques such as voting or averaging to improve the final decision [13]. Ensemble algorithms can be categorized into three branches: boosting [14], stacking [15], and bagging [16]. Stacking ensemble is considered as the best technique for building ensemble models because it is based on a meta-learner, which learns from data how to weight the base classifiers and combine them in the best way to optimize the performance of the resulting model. Ensemble stacking optimizes a set of heterogeneous base models and combines their decisions using a meta-learner [15].

In this study, we proposed an optimized ensemble stacking model that merged the two pre-trained hybrid models of CNN-LSTM and CNN-GRU with a meta-learner (SVM) to enhance the performance of heart disease prediction. In addition, Recursive Feature Elimination (RFE) has been used to choose the most informative features from two heart disease datasets. Our contributions can be summarized as follows:

- We proposed two hybrid models with heterogeneous architectures: CNN-LSTM and CNN-GRU were proposed and optimized.
- We proposed a stacking ensemble model that merged the previous pre-trained hybrid models of CNN-LSTM and CNN-GRU. The best meta-learner classifier has been selected based on the experimental results. The SVM algorithm achieved the best results as the meta-classifier to determine the best weights of the base classifiers;
- We compared the proposed model with different ML models using two benchmark heart disease datasets;
- The proposed model significantly outperformed all other models and achieved the best results.

The remainder of the paper is structured as follows: Section 2 discussed heart disease-related works. The section describes the main phases and approaches Section 3 of predicting heart disease. Section 4 describes the results and discussion results. Finally, the paper is concluded in Section 5.

## 2. Related Work

Machine learning and deep learning have been used to predict heart disease. For example Kavitha M. et al. [17] suggested a hybrid model that combines DT and RF to predict heart disease using the Cleveland dataset. They contrasted the hybrid model’s performance with that of DT and RF. Ishaq A. et al. [18] applied different ML algorithms: SVM, DT, LR, NB, Adaptive boosting (AdaBoost), Stochastic Gradient Descent (SGD), RF, Gradient Boosting Machine (GBM), and Extra Tree Classifier (ETC) using the Cleveland heart disease dataset to analyzes the heart failure. The results showed that ETC gave the best performance and outperformed other models. Ansarullah, S.I., et al. [19] used ML algorithms to predict heart disease: NB, RF, DT, K-NN, and SVM. The dataset was gathered in Kashmir from many heterogeneous data sources (India). The results showed that RF has the best model performance.

Many authors applied feature selection methods with ML and DL models to predict heart disease. For example, Spencer R. et al. [20] used Chi2, ReliefF, symmetrical uncertainty (SU), and PCA feature selection methods to extract the important features from four heart-disease datasets. They applied BayesNet, Logistic, Stochastic Gradient Descent (SGD), and KNN Adaboost to the full and selected features. The result showed that the BayesNet model was recorded as the best performer using the Chi-2 feature selection compared with other models. Bharti R. et al. [21] used the Lasso algorithm to select features from the heart disease dataset. They applied ML and DL models: LR, KNN, SVM, RF, DT, and ANN, respectively. The results showed that ANN has the best performance compared to ML models. Gokulnath, C.B., et al. [22] used KNN, MLP, SVM, and J48 for heart disease detection. The datasets were gathered from a variety of sources. The authors applied various feature selection strategies, including the extra tree classifier, gradient boosting classifier, random forest, recursive feature removal, and XG boost classifier. In the study by Amin, M.S., et al. [23], in order to increase the prediction accuracy, the authors proposed a voting hybrid model based on NB and LR. They used k-NN, DT, NB, LR, SVM, Neural Network (NN), and the hybrid model to choose meaningful characteristics from the Cleveland heart disease dataset. The hybrid model was given the best performance compared to other models. Bashir S. et al. [24] used DT, LR, NB, SVM, and RF models with feature extraction methods with the Cleveland heart disease dataset to predict heart disease. The results showed that LR and SVM with feature selection methods had better accuracy than the other models. Javid I. et al. [25] developed model-based GRU and RF (GRU-RF) for heart disease detection. The GRU-RF was compared with RF, GRU, KNN, and DNN algorithms and achieved the best performance. Chae M. et al. [26] proposed a hybrid model, LSTM–GRU, and compared it with DT, RF, LR, LSTM, and GRU to predict heart disease. They used the dataset from Soonchunhyang University Cheonan Hospital in Korea to train and test the models. They improved the performance models based on hyperparameter adjustment, the quantity of primary patient data, and input parameters. The results indicate that when compared to other models, the GRU model outperforms the others. In the study by Narmadha, S. et al. [27], the authors used LSTM and GRU hyperparameter tuning to enhance the performance of the algorithms. The outcomes demonstrated that the GRU provides better accuracy than the LSTM across the board.

The authors have used ensemble models to predict heart disease. For example, Adhikari, B. et al. [28] applied LR, SVM, DT, K-NN, GNB, and ensemble models using a dataset collected from the UCI heart disease dataset. They used the voting and averaging ensemble models built by combining the ML above models. The results showed that the ensemble model was the best performer compared with other models. Javid, I. et al. [29] used RF, SVM, K-NN, LSTM, Hard Voting Ensemble Model, and GRU for heart disease prediction. The results showed that the Hard Voting Ensemble Model recorded higher accuracy compared to other models.

Ghosh P. et al. [30] proposed hybrid models that integrated boosting and bagging with traditional ML models: KNN, DT, and RF. The hybrid models: K-NN Bagging Method (KNNBM), DT-Bagging Method (DTBM), AdaBoost (AB), and Random Forest Bagging Method (RFBM) were applied to heart disease datasets. Relief, Least Absolute Shrinkage, and Selection Operator were the three feature selection approaches they used (LASSO). When compared to other models, the RFBM model showed the best performance.

Previous studies do not use ensemble stacking based on heterogeneous hybrid deep learning models to predict heart disease. In addition, most previous studies have used the Cleveland heart disease database to perform this experiment. In our work, we used a new large heart disease dataset, and we proposed ensemble stacking models based on optimizing different heterogeneous hybrid models: CNN-LSTM and GRU-LSTM.

## 3. Methodology

**Figure 1:**
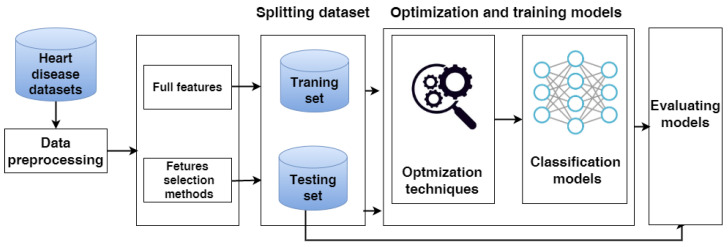
The phases of predicting heart disease.

In this study, we evaluate three approaches: the classical machine learning approach, the hybrid models approach, and a proposed model. These models are applied to the full feature set and selected feature set. The proposed model for predicting heart disease has several steps including data collection, data preprocessing, data splitting, feature selection, and evaluation models, as shown in Figure 1. Each phase is described in detail as follows.

### 3.1. Heart Disease Datasets

In our work, we used two heart disease datasets.

#### 3.1.1. Dataset 1

We used the large heart disease dataset (Heart Disease) [31]. This data includes 18 independent features and one dependent variable as the class label for predicting heart disease. The class label includes two values: 0 represents the healthy class label, and 1 represents the heart disease class label. Table 1 presents the number of medical records for each class in the training and testing sets. The description of each feature is described in a Supplementary File.

#### 3.1.2. Cleveland Dataset

The Cleveland dataset [32] includes 13 independent variables as features and one dependent variable as the class label used to diagnose heart disease. The class label includes two values: 0 represents the healthy class label, and 1 represents the heart disease class label. Table 1 presents the number of medical records for each class in the training and testing sets of the Cleveland heart disease dataset. The description of each feature is described in the Supplementary File.

### 3.2. Data Pre-Processing

The first heart disease dataset includes 14 numeric features and four categorical features. The data was preprocessed after collection as follows: removing duplicate records and encoding category data into numerical data such as smoking and skin cancer.

### 3.3. Data Splitting

The two datasets are divided into two sets using a stratified sampling method: 80% training sets and 20% testing sets. Models are trained and optimized using training data. The test set is employed to assess and test the model. The stratified sampling method is one way of splitting the dataset used to get samples that accurately reflect the distribution of classes in the population. It separates the dataset into homogeneous subsets; each subset contains the same percentage of every class [33,34]. This method has been used in studies of different fields of healthcare [35,36,37].

### 3.4. Feature Selection Methods

In our work, we use the Recursive Feature Elimination (RFE) feature selection method to extract the most informative features from each dataset. The RFE determines the essential features by figuring a high correlation between features and the target [38]. It assigns one value as ranking for features if the features have high collaboration with the target. A novel RFE strategy is recently presented that used RF and SVM to evaluate features rather than classification performance and selects the minor significant features for deletion [39,40].

### 3.5. Machine Learning Approach

#### 3.5.1. ML Algorithms

We tested many classical ML models from different families including SVM [41,42,43,44], Logistic Regression (LR) [45,46], Nave Bayes (NB) [47], Decision tree (DT) [48], Random Forest (RF) [49,50], and K-nearest Neighbors (k-NN) [51].

#### 3.5.2. Optimization Techniques for Classical Models

Grid search is employed to fine-tune hyperparameters of different classical ML models by generating discrete grids within the hyperparameter domain and select the list of parameters that give the best performance [52]. Data is split into two segments using the cross-validation technique: one is used to train and validate the models (training set), and the other is utilized for model testing (testing set) [19]. The training set has been used to validate the models using the k-fold cross validation technique.

### 3.6. The Hybrid Models

#### 3.6.1. The Hybrid Model Architectures

**Figure 2:**
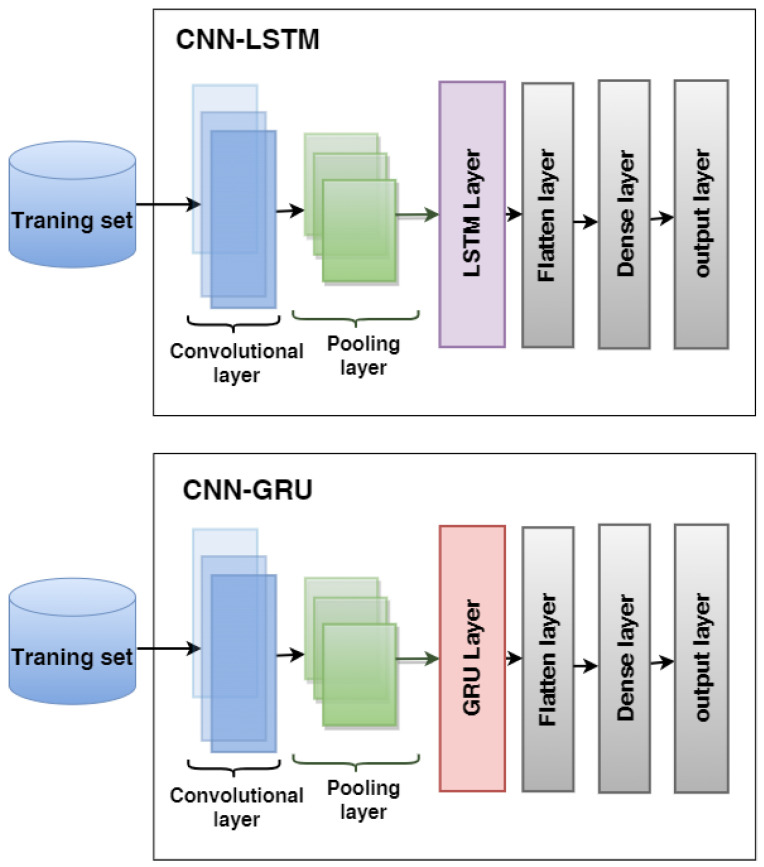
The architecture of the hybrid models CNN-LSTM and CNN-GRU used to predict heart disease.

We proposed two hybrid models: CNN-LSTM and CNN-GRU for predicting heart disease. The structures of hybrid models are illustrated in Figure 2.

- The first model is CNN-LSTM, which combines CNN with LSTM, consisting of a convolutional layer, a max-pooling layer, an LSTM layer, a flatten layer, a fully connected, and an output layer;
- The second model is CNN-GRU, which combines CNN with GRU. The architecture consists of a convolutional layer, a max-pooling layer, an GRU layer, a flatten layer, a fully connected, and an output layer.

#### 3.6.2. Optimization Techniques for Hybrid Models

The Bayesian optimizer is used to optimize the hybrid models. This search technique quickly generates the search space and locates the best hyperparameter values for the models [53]. We adopt the parameter settings for CNN-LSTM and CNN-GRU, as shown in Table 2.

### 3.7. The Proposed Stacking Ensemble Model

**Figure 3:**
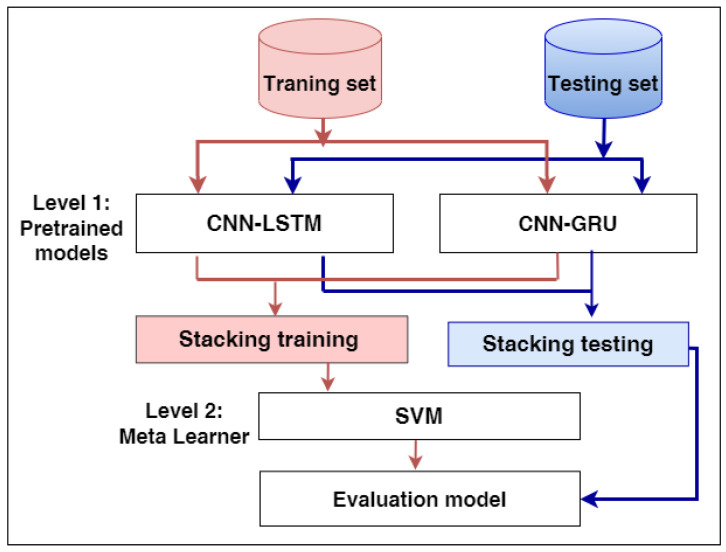
The proposed model for predicting heart disease.

In this work, our model is developed using two levels: Level-1 and Level-2, as shown in Figure 3. Level-1 begins by loading the pre-trained models of hybrid models CNN-LSTM and CNN-GRU, and the layers of the models are frozen except for the last layers. The models anticipate the training set’s output probabilities and subsequently integrate them into stacking training. Secondly, the models estimate the output probabilities of the testing set and aggregate them in stacking testing. At Level 2, SVM, as a meta-learner, is trained and optimized using stacking training and Grid search, respectively, while producing the final results using stacking testing.

### 3.8. Evaluating Models

The metrics for classification performance that are most frequently employed are accuracy (ACC), precision (PRE), recall (REC), and *F*1-score (*F*1). In contrast to the True Positive (*TP*), which denotes that the person is ill and the test is positive, the True Negative (*TN*) shows that the person is healthy and the result is negative. False positives are tests that come back positive even when the subject is healthy (*FP*). When a test is negative, but the subject is ill, it is known as a false negative (*FN*).

(1) $$Accuracy=\frac{TP+TN}{TP+FP+TN+FN}.$$

(2) $$Precision=\frac{\mathrm{TP}}{\mathrm{TP}\;+\;\mathrm{FP}}$$

(3) $$Recall=\frac{TP}{TP+FN}$$

(4) $$F1-score=\frac{2\cdot precision\cdot recall}{precision+recall}$$

## 4. Experimental Results

In this section, we describe the rank of features after applying the RFE to the two datasets. Moreover, we describe the results of the performance of using ML models (SVM, LR, RF, NB, and KNN), the hybrid models (CNN-LSTM, CNN-GRU), and the proposed model to full and selected features.

### 4.1. Experimental Setup

The experiments in this paper are implemented using Google Colab with Python libraries such as Scikit-learn, TensorFlow, and others. We used grid-search and the Bayesian optimizer to optimize the ML and hybrid models. We used RFE technique to identify the best features from the two datasets. The two datasets are separated into two sets: 80% training and 20% testing set using the stratified methods. The models are trained and tested by utilizing the training and testing sets, respectively.

### 4.2. Results of Dataset1

#### 4.2.1. Feature Selection Results

**Figure 4:**
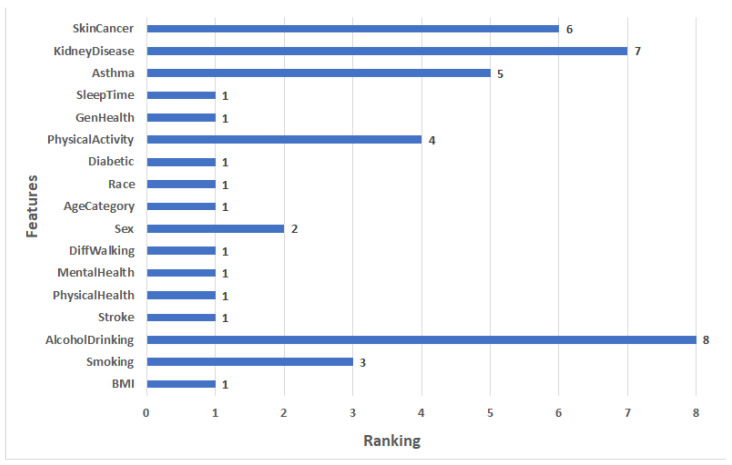
The ranking features for heart disease Dataset 1.

In the experiments, we used the RFE to extract the important features from the heart disease dataset by assigning ranking for every feature. The critical features are ranked 1, and the least important features are ranked 8. The features ranking is shown in Figure 4. We can see that the most significant 10 features have a ranking of 1: BMI, Stroke, PhysicalHealth, MentalHealth, DiffWalking, AgeCategory, Race, Diabetic, GenHealth, and SleepTime. The lowest important feature has a ranking of 8, which is AlcoholDrinking.

#### 4.2.2. Results of Applying Models

This section presents the ACC, PRE, REC, and F1 of ML, hybrid models, and the proposed model for Dataset 1. In the hybrid models CNN-LSTM and CNN-GRU some parameters were adapted: batch_size of 500, epoch = 50, learning rate = 0.00004, and the optimizer used is Adam. Some of the best values of CNN-LSTM and CNN-GRU hyperparameters that were selected by KerasTuner are shown in Table 3.

Table 4 shows the results of applying ML, hybrid models, and the proposed model with full features and selected features by RFE to the heart disease Dataset 1.

- Results of the full features:
  For ML models, RF and LR register approximately the same highest scores (75.32% of ACC, 75.44% of PRE, 75.32% of REC, 75.33% of F1) and (75.60% of ACC, 75.60% of PRE, 75.60% of REC, 75.60% of F1), respectively. NB records the worst scores (60.87% of ACC, 64.98% of PRE, 60.87% of REC, 56.69% of F1). KNN registers the second-highest scores (73.16% of ACC, 73.47% of PRE, 73.16% of REC, 73.16% of F1).
  For hybrid models, CNN-LSTM has the highest scores (76.64% of ACC, 76.9% of PRE, 76.64% of REC, and 76.65% of F1). CNN-GRU records the lowest scores (75.63% of ACC, 75.65% of PRE, 75.63% of REC, 75.58% of F1).
  The proposed model records the highest scores (ACC = 78.81%, 78.1% of PRE, 78.81% of REC, and 78.81% of F1) compared to other models. It improves ACC by 2.17, PRE by 1.2, REC by 2.17, and F1 by 2.16 compared to CNN-LSTM.
- Results of the selected features:
  For ML models, RF and LR register approximately the same highest scores (73.02% of ACC, 73.06% of PRE, 73.02% of REC, 73.03% of F1) and (73.58% of ACC, 73.60% of PRE, 73.58% of REC, = 73.59% of F1), respectively. NB records the worst scores (60.84% of ACC, 64.97% of PRE, 60.84% of REC, F1 = 56.63%). KNN registers the second-highest scores (72.59% of ACC, 72.92% of PRE, 72.59% of REC, F1 = 72.59%).
  The top scores for hybrid models belong to CNN-LSTM (75.22% of ACC, 75.42% of PRE, 75.22% of REC, and 75.22% of F1). The lowest scores are recorded by CNN-GRU (74.07% of ACC, 74.23% of PRE, 74.07% of REC, and 74.08% of F1).
  In comparison to other models, the proposed model achieves the greatest scores (77.42% of ACC, 77.99% of PRE, 77.42% of REC, and 77.39% of F1). In comparison to CNN-LSTM, it enhances ACC by 2.2%, PRE by 2.57%, REC by 2.2%, and F1 by 2.17%.

### 4.3. Results of the Cleveland Dataset

#### 4.3.1. Feature Selection Results

**Figure 5:**
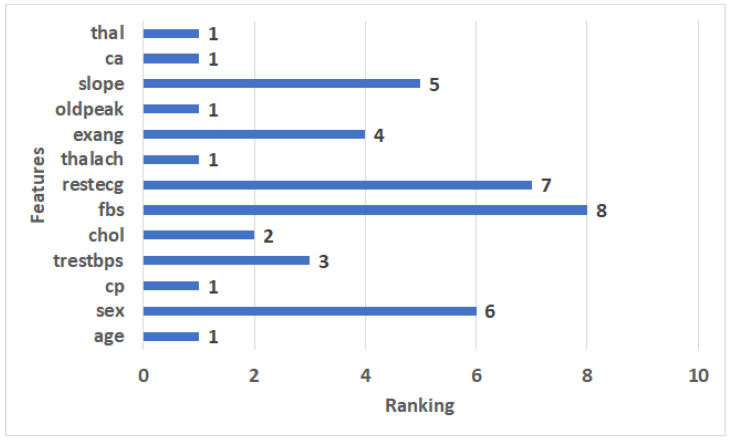
The ranking features for the Cleveland dataset.

In the experiments, we used the RFE to extract the important features from the Cleveland dataset. It assigns features a value of ranking, with the critical features having a ranking of 1, and the least important features having a ranking of 8. The features ranking is shown in Figure 5. We can see that the 8 most significant features have a ranking of 1: age, cp, thalach, oldpeak, ca, and thal. The least important feature has a ranking of 8, which is fbs.

#### 4.3.2. Results of the Applied Models

This section presents the setting of values parameters for models and the results of applied ML, hybrid models, and the proposed model with the full and selected features for the Cleveland dataset. The following settings were modified for CNN-LSTM and CNN-GRU hybrid models: batch size = 50, epoch = 50, learning rate = 0.00004, and the optimizer used is Adam. Some of the best CNN-LSTM and CNN-GRU hyperparameter values as determined by KerasTuner are shown in Table 5.

Table 6 shows the results of applying ML, hybrid models, and the proposed model with full features and selected features by RFE to the Cleveland dataset.

- Full features
  For ML models, RF has the highest scores (86.34% of ACC, 86.34% of PRE, 86.34% of REC, and 86.34% of F1). NB records the lowest scores (60.00% of ACC, 60.05% of PRE, 60.00% of REC, 59.74% of F1). DT registers the second-highest scores (82.44% of ACC, 82.46% of PRE, 82.44% of REC, 82.44% of F1).
  For hybrid models, CNN-LSTM has the highest scores (89.76% of ACC, 89.96% of PRE, REC = 89.76% of REC, F1 = 89.75%). CNN-GRU records the lowest scores (88.29% of ACC, 89.06% of PRE, REC = 88.29% of REC, 88.26% of F1).
  The proposed model records the highest scores (97.17% of ACC, 97.42% of PRE, 97.17% of REC, 97.15% of F1) compared to the other models. It improves ACC by 7.41, PRE by 7.46, REC by 7.41, and F1 by 7.4 compared to CNN-LSTM.
- Selected features
  For ML models, RF has the highest scores (82.93% of ACC, 82.99% of PRE, 82.93% of REC, 82.91% of F1). NB records the lowest scores (64.88% of ACC, 64.90% of PRE, 64.88% of REC, 64.88% of F1). DT registers the second-highest scores (81.95% of ACC, PRE = 82.01%, 81.95% of REC, 81.93% of F1).
  For hybrid models, CNN-LSTM has the highest scores (86.34% of ACC, 86.41% of PRE, 86.34% of REC, and 86.34% of F1). CNN-GRU records the lowest scores (85.85% of ACC, 86.92% of PRE, 85.85% of REC, 85.78% of F1).
  The proposed model records the highest scores (91.22% of ACC, 91.29% of PRE, 91.22% of REC, 91.22% of F1) compared to other models. It improves ACC by 4.88, PRE by 4.88, REC by 4.88, and F1 by 4.88 compared to CNN-LSTM.

### 4.4. Discussion

We used two heart disease datasets downloaded from Kaggle. We applied RFE feature selection methods to select the essential features. The proposed model, in all cases, has achieved the highest score compared with the other models.

#### 4.4.1. Dataset1

**Figure 6:**
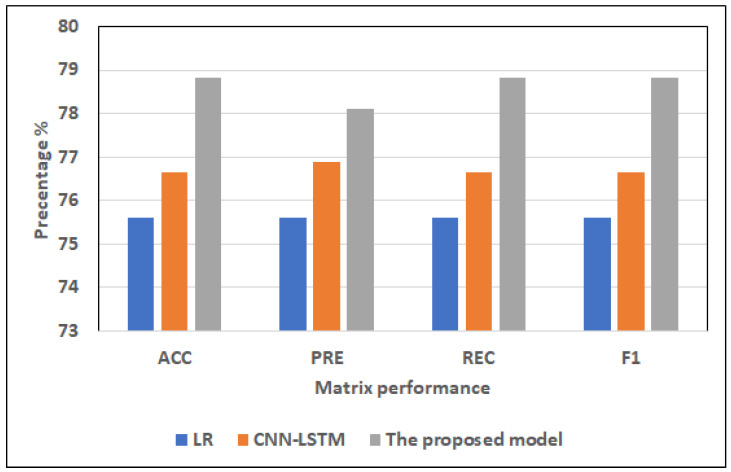
The best models for applying models with full features for Dataset 1.

**Figure 7:**
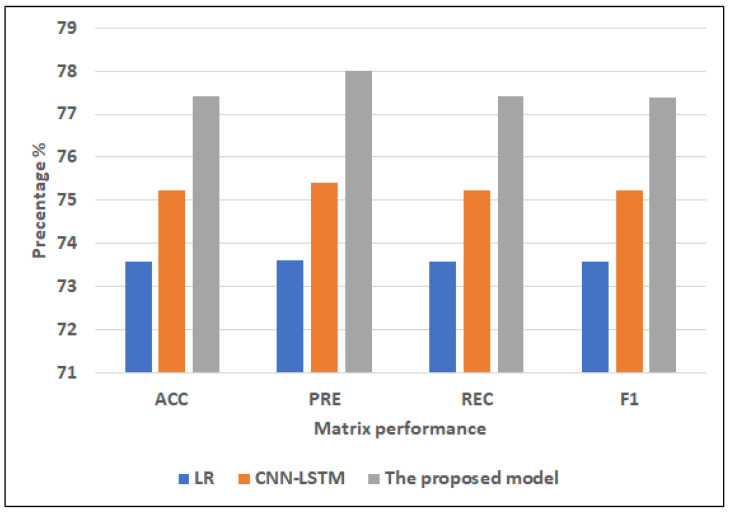
The best models for applying models with selected features for Dataset 1.

Figure 6 and Figure 7 show the best models for applying models with full features and selected features. We can see that the proposed model has achieved the highest scores with full features at ACC = 78.81%, PRE = 78.81%, REC = 78.81%, and F1 = 78.81% compared to other models with full features and selected features, and It improves ACC by 2.17, PRE by 1.2, REC by 2.17, and F1 by 2.16 compared to CNN-LSTM. In addition, it has the highest scores with selected features at (ACC = 77.42%, PRE = 77.99%, REC = 77.42%, F1 = 77.39%, and it improves ACC by 2.2%, PRE by 2.57%, REC by 2.2%, and F1 by 2.17%. LR has the lowest scores with full features and selected features.

#### 4.4.2. Cleveland Dataset

**Figure 8:**
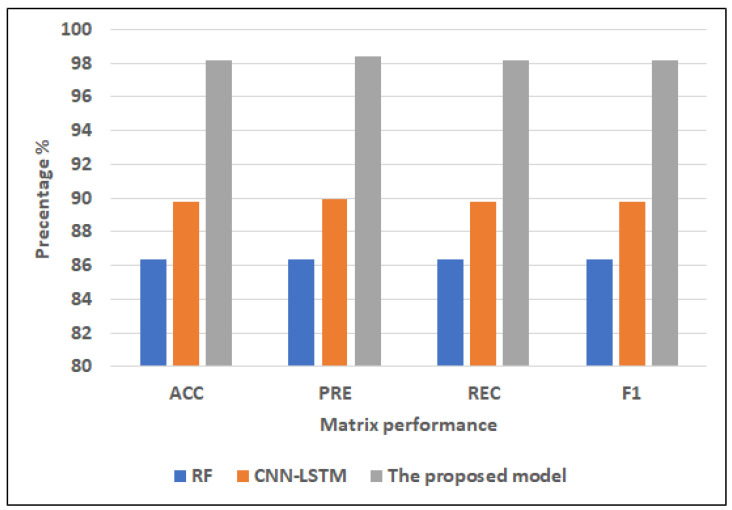
The best models for applying models with full features for Dataset 2.

**Figure 9:**
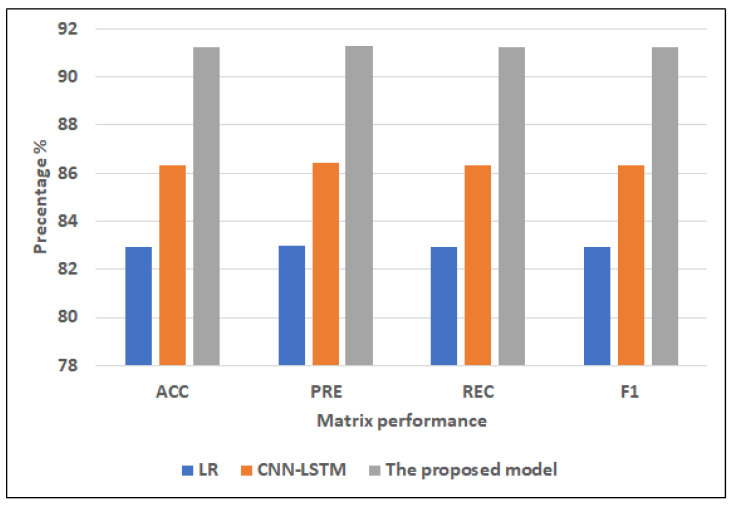
The best models for applying models with selected features for Dataset 2.

Figure 8 and Figure 9 show the best models for applying models with full features and selected features. We can see that the proposed model has achieved the highest scores with full features at ACC = 98.17%, PRE = 98.42%, REC = 98.17%, and F1 = 98.15% compared to other models with full features and selected features, and it improves ACC by 3.41, PRE by 3.46, REC by 3.41, and F1 by 3.4 compared to CNN-LSTM. In addition, it has the highest scores with selected features at (ACC = 91.22%, PRE = 91.29%, REC = 91.22%, F1 = 91.22%, and it improves ACC by 4.88, PRE by 4.88, REC by 4.88 and F1 by 4.88 compared to CNN-LSTM. RF has the lowest scores with full features, and LR has the lowest scores with the selected features.

#### 4.4.3. Comparison with Literature Studies

By assessing the developed model against the current models we could observe that our approach enhanced the scores more than other models. We compared our approach with the approach by authors who used the Cleveland Dataset, as shown in Table 7. The authors of Ref. [17] used a hybrid model combining DT and RF, which recorded 88.7% of ACC. The authors in Refs. [20,22,23,24,29], used various models, none of which were accurate to more than 90%, which recorded 85%, 88.34%, 87.41%, 84.85%, and 85.71%, respectively. While in Refs. [18,21,28], the authors achieved an accuracy of over 90%. The proposed model has achieved the highest ACC at 98.41% compared to the ACC values in these studies.

## 5. Conclusions

The study proposed a deep staking ensemble to improve the performance of heart disease prediction. The proposed model was based on the integration of two pre-trained and optimized deep hybrid models: CNN-LSTM and CNN-GRU. The SVM classifier has been used as the meta-learner model. The first hybrid model was the CNN-LSTM model, which combined CNN and LSTM layers. The second hybrid model was the CNN-GRU model, which combined CNN with GRU models. RFE was used to choose the most important features from two heart disease datasets. The proposed models were compared with five classical ML models, including LR, RF, K-NN, DT, NB, and hybrid models (i.e., CNN-LSTM and CNN-GRU). Results were collected with the full feature set and a selected feature set. Compared to other models, the result generated by the proposed model had the optimum performance with all the features. For the first dataset, the proposed model had the highest ACC of 78.81%, PRE of 78.1%, REC of 78.81%, and F1 of 78.81. For the Cleveland dataset, the proposed model had the highest ACC of 97.17%, PRE of 97.42%, REC of 97.17%, and F1 of 97.15%. In addition, the proposed model achieved better results than the literature. As a result, the proposed model can improve the disease prediction and can improve the quality of life of the heart disease patients. In the future, we will test the performance of the proposed model with other datasets. We will extend the model by adding other modalities such as images and EEG data. We will provide interpretability features to the proposed model.

## Figures and Tables

**Table 1:** The number of medical records for each class in the heart disease datasets.

Dataset	Classes	Training Set	Testing Set	Total
Dataset 1	Heart disease	21,898	5475	27,373
	Healthy	24,000	6000	30,000
	Total	45,898	11,475	57,373
Cleveland Dataset	Heart disease	421	105	526
	Healthy	399	100	499
	Total	820	205	1025

**Table 2:** Setting values of the parameters.

Parameters	Values
filters	[16,128]
Kernel_size	[2,3,4,5]
Pool_Size	[2,3,4,5]
Unit_LSTM	between 20 and 500
Unit_GRU	between 20 and 500
Unit_Dense	between 20 and 500

**Table 3:** The best values of the parameters for CNN-LSTM and CNN-GRU.

Dataset	Models	Parameters	Full Features	Selected Features
Dataset 1	CNN-LSTM	filters	128	16
		Kernel_size	4	4
		Pool_Size	2	2
		Unit_LSTM	380	40
		Unit_Dense	140	50
	CNN-GRU	filters	128	16
		Kernel_size	4	4
		Pool_Size	2	2
		Unit_GRU	100	320
		Unit_Dense	100	200

**Table 4:** Result of applying models with full features and the selected features for Dataset 1.

Approaches	Models	Features	Matrix Performance			
			ACC	PRE	REC	F1
Regular ML approach	RF	Full features	75.32	75.44	75.32	75.33
		Selected features	73.02	73.06	73.02	73.03
	LR	Full features	75.60	75.60	75.60	75.60
		Selected features	73.58	73.60	73.58	73.59
	DT	Full features	67.28	67.26	67.28	67.27
		Selected features	65.76	65.76	65.76	65.7
	NB	Full features	60.87	64.98	60.87	56.69
		Selected features	60.84	64.97	60.84	56.63
	KNN	Full features	73.16	73.47	73.16	73.16
		Selected features	72.59	72.92	72.59	72.59
The hybrid models	CNN-LSTM	Full features	76.64	76.9	76.64	76.65
		Selected features	75.22	75.42	75.22	75.22
	CNN-GRU	Full features	75.63	75.65	75.63	75.58
		Selected features	74.07	74.23	74.07	74.08
The proposed model	Stacking SVM	Full features	78.81	78.1	78.81	78.81
		Selected features	77.42	77.99	77.42	77.39

**Table 5:** The best values of the parameters for the Cleveland dataset.

Datasets	Models	Parameters	Full Features	Selected Features
Cleveland dataset	CNN-LSTM	filters	128	16
		Kernel_size	4	5
		Pool_Size	2	2
		Unit_LSTM	360	60
		Dense Unit	160	20
	CNN-GRU	filters	64	16
		Kernel_size	4	5
		Pool_Size	2	2
		Unit_GRU	440	80
		Unit_Dense	160	40

**Table 6:** Result of applying models with full features and selected features for the Cleveland dataset.

Approaches	Models	Features	Matrix Performance			
			ACC	PRE	REC	F1
Regular ML approach	RF	Full features	86.34	86.34	86.34	86.34
		Selected features	82.93	82.99	82.93	82.91
	LR	Full features	67.32	67.43	67.3	67.18
		Selected features	73.17	73.19	73.17	73.14
	DT	Full features	82.44	82.46	82.44	82.44
		Selected features	81.95	82.01	81.95	81.93
	NB	Full features	60.00	60.05	60.00	59.74
		Selected features	64.88	64.90	64.88	64.88
	KNN	Full features	60.00	60.25	60.00	59.92
		Selected features	66.34	66.62	66.34	66.29
The hybrid models	CNN-LSTM	Full features	89.76	89.96	89.76	89.75
		Selected features	86.34	86.41	86.34	86.34
	CNN-GRU	Full features	88.29	89.06	88.29	88.26
		Selected features	85.85	86.92	85.85	85.78
The proposed model	Stacking SVM	Full features	97.17	97.42	97.17	97.15
		Selected features	91.22	91.29	91.22	91.22

**Table 7:** Comparison between previous studies and the proposed model for the Cleveland dataset.

Papers	Models	Datasets	Accuracy
[17]	hybrid model that combines DT and RF	Cleveland Dataset.	88.7%
[18]	DT, AdaBoost, LR, SGD, RF, GBM, ETC, GNB, SVM	Cleveland Dataset.	92.62%
[20]	BayesNet, LR, SGD, IBK(k = 21), AdaB(DS), AdaB(Logistic), RF	Cleveland Dataset.	85%
[21]	LR, KNN, SVM, RF, DT, DL	Cleveland Dataset.	94.2%
[22]	KNN, MLP, SVM, and J48	Cleveland Dataset.	88.34%
[23]	K-NN, DT, NB, LR, SVM, NN, Vote	Cleveland Dataset.	87.41%
[24]	DT, LR, RF, NB, LR (SVM)	Cleveland Dataset.	84.85%
[28]	Ensemble Voting,	Cleveland Dataset.	96.43%
[29]	Hard Voting Ensemble Model	Cleveland Dataset.	85.71%
Our work	The proposed model	Cleveland Dataset.	98.41

## Data Availability

The direct link in the dataset citations will take you to all of the datasets that were utilized to support the study’s assertions.

## Supplementary Materials

The following supporting information can be downloaded at: https://www.mdpi.com/article/10.3390/diagnostics12123215/s1, Table S1: Features information and description of heart disease dataset1; Table S2: Features information and description of Cleveland heart disease dataset 2016.
