# Supplementary material for: Ensemble Learning Based on Hybrid Deep Learning Model for Heart Disease Early Prediction
Source: Diagnostics (Basel). 2022 Dec 18;12(12):3215. doi: 10.3390/diagnostics12123215 (PMC9777370; doi:10.3390/diagnostics12123215)
Supplement: Supplementary file 1 [file diagnostics-12-03215-s001.zip › diagnostics-2044686-supplementary.pdf]

Table S1: Features information and description of heart disease dataset1

SR NO.	Attribute	Description	Values	Data Type
1	HeartDisease	suffering from heart disease, no heart disease	Yes - No	Categorical
2	BMI	Body mass index	12.02 -94.85	Numerical
3	Smoking	Patient's smoking status	Yes - No	Categorical
4	AlcoholDrinking	Drinking wine(adult men: more than 14 drinks per week, adult women: more than7 drinks per week)	Yes - No	Categorical
5	Stroke	suffered a stroke, no a stroke	Yes - No	Categorical
6	PhysicalHealth	How many times have you had poor physical health in the past 30 days?	0-30 days	Numerical
7	MentalHealth	Number of days mental health is not good per the past 30days	0-30 days	Numerical
8	DiffWalking	Do you have serious difficulty climbing stairs or walking?	Yes - No	Categorical
9	Sex	Gender of patient	Female, Male	Categorical
10	AgeCategory	13-level age group	18-24, 25-29,30-34, 35-39,40-44,45-49, 50-54,55-59, 60-64, 65-69, 70-74, 75-79,80 or older	Categorical
11	Race	Imputed ethnicity /race value.	Black, Other, Asian, American Indian/Alaskan Native, White, Hispanic	Categorical
12	Diabetic	Do you have diabetes or not?	Yes - No	Categorical
13	PhysicalActivity	Adults who did exercise and physical activity in the past 30 days, except for their usual work.	Yes - No	Categorical
14	GenHealth	Assessment of the patient's health status in general	Poor, Fair, Good, Very good, Excellent	Categorical
15	SleepTime	How many hours of sleep do you get in 24 hours?	1-24 hour	Numerical
16	Asthma	Do you have asthma or not?	Yes - No	Categorical
17	KidneyDisease	Do you have kidney disease except kidney stones, bladder infection or incontinence?	Yes - No	Categorical
18	SkinCancer	Do you have skin cancer or not?	Yes - No	Categorical

Table S2: Features information and description of Cleveland heart disease dataset 2016

S.no	Feature name	Feature	code Description	Domain of values (min-max)
1	Age	AGE	Age in years	30 < age < 77
2	Sex	SEX	Male = 1<br>Female = 0	1<br>0
3	Type of chest pain	CPT	1 = atypical angina<br>2 = typical angina<br>3 = asymptomatic	1<br>2<br>3
4	Resting blood pressure	RBP	mm Hg admitted at the hospital	94–200
5	Serum cholesterol	SCH	In mg/dl	120–564
6	Fasting blood sugar >120 mg/dl	FBS	Fasting blood sugar >120 mg/dl (1 = true; 0 = false)	1<br>0
7	Resting electrocardiographic results	RES	1 = having ST-T<br>2 = hypertrophy	1<br>2
8	Maximum heart rate achieved	MHR	-	71-202
9	Exercise-induced angina	EIA	1 = yes<br>0 = no	1<br>0
10	Old peak = ST depression induced by exercise relative to rest	OPK	-	0–6.2
11	Slope of the peak exercise ST segment	PES	1 = up sloping<br>2 = flat2	1<br>2

			3 = down sloping	3
12	Number of major vessels (0–3) colored by fluoroscopy	VCA	-	1<br>2<br>3
13	Thallium scan	THA	3 = normal<br>6 = fixed defect<br>7 = reversible defect	3<br>6<br>7
